# The impact of ward nighttime noise management under the enhanced recovery after surgery protocol on postoperative sleep quality and mental health in patients with lower extremity fractures: A retrospective, non-randomized, historically controlled study

**DOI:** 10.12669/pjms.42.6.15669

**Published:** 2026-06

**Authors:** Ruzhen Zhang, Cui Huang

**Affiliations:** 1 Ruzhen Zhang, Department of Traumatology and Orthopedics, The Fourth Hospital of Wuhan, Wuhan, Hubei Province 430000, P.R. China; 2 Cui Huang, Department of Traumatology and Orthopedics, The Fourth Hospital of Wuhan, Wuhan, Hubei Province 430000, P.R. China

**Keywords:** Enhanced recovery after surgery, Lower extremity fractures, Mental health, Nighttime noise management, Sleep quality

## Abstract

**Objective:**

To explore the impact of ward nighttime noise management under the enhanced recovery after surgery (ERAS) protocol on postoperative sleep quality and mental health in patients with lower extremity fractures.

**Methodology:**

This was a retrospective, non-randomized, historically controlled study conducted at Gutian Campus of our Hospital. It included 65 patients with lower extremity fractures who received ward nighttime noise management under the ERAS protocol between April 2024 to April 2025 (observation group), and 65 patients who received conventional nursing management between April 2023 to March 2024 served as the control group. The two groups were matched at a 1:1 ratio based on gender, age, and American Society of Anesthesiologists (ASA) classification. The primary outcome was the scores of Pittsburgh Sleep Quality Index (PSQI). Secondary outcomes included Self-rating Depression Scale (SDS), Self-rating Anxiety Scale (SAS) scores, Generic Quality of Life Inventory-74 (GQOL-74), and nursing satisfaction.

**Results:**

During the perioperative period, the noise level in the observation group was significantly lower than that in the control group (*P*<0.05). At 7-days postoperatively, the PSQI, SDS, and SAS scores in the observation group were significantly lower than those in the control group, while the improved scores of social function, psychological function, and physical function were significantly higher (all *P*<0.05). The nursing satisfaction in the observation group was significantly higher than that in the control group (*P*<0.05).

**Conclusions:**

Ward nighttime noise management under the ERAS protocol is associated with improved postoperative sleep and mental health of patients with lower limb fractures.

## INTRODUCTION

The concept of Enhanced Recovery After Surgery (ERAS) aims to optimize various perioperative management measures, mitigate surgical stress, lower the incidence of complications, and promote the rapid postoperative recovery of patients.^1^,^2^ During the perioperative period, ERAS emphasizes multidisciplinary collaboration, aiming to provide patients with comprehensive treatment and nursing plans.^3^ Currently, ERAS has been widely applied in the field of orthopedic surgery.^3^,^4^ A meta-analysis conducted by Zhang et al.^4^, which included 44 studies, indicated that ERAS can significantly alleviate pain and reduce the incidence of complications in elderly patients undergoing joint replacement surgery.

Sleep is a critical factor influencing patients’ postoperative recovery. Poor sleep quality is prone to triggering negative emotions such as anxiety, depression, and irritability in patients, increasing their psychological burden and thereby hindering the postoperative rehabilitation process.^5^,^6^ However, excessively high nighttime noise levels in wards frequently serve as a major factor disrupting patients’ sleep.^7^ The World Health Organization (WHO) recommends that ward nighttime noise should not exceed 30 dB, but actual measurements have shown that nighttime noise in many wards frequently reaches 50 dB or even higher.^8^,^9^ The main sources of these noises include medical equipment alarms, conversations among medical staff, activities of patients and their family members, and traffic noise outside the wards.^7^–^9^

Patients with lower extremity fractures are inherently susceptible to sleep problems and psychological stress due to limited limb mobility, pain, and other factors.^10^ Excessive postoperative nighttime noise levels will further exacerbate their physical and psychological discomfort, and adversely affect the rehabilitation process.^10^,^11^ Therefore, reducing ward nighttime noise levels and creating a quiet, comfortable sleep environment are of great significance for the postoperative recovery of patients with lower extremity fractures.

Since April 2024, the Department of Orthopedics at Gutian Campus of our Hospital has implemented nighttime noise management based on the ERAS protocol in general wards and formulated relevant standardized regulations. The nighttime noise management involves not only sound control, but also behavioral regulation, adjustment of nursing rhythm, caregiver management, patient comfort support, and ward order maintenance. This study explored the impact of nighttime noise management based on the ERAS protocol on the postoperative sleep quality and mental health of patients with lower extremity fractures. Our goal was to improve the overall service quality and management level of the hospital.

## METHODOLOGY

This was a retrospective, non-randomized, historically controlled study conducted at Gutian Campus of our Hospital. We retrospectively reviewed the records of patients with lower extremity fractures who underwent surgical treatment in the Department of Orthopedics of our hospital between April 2023 and April 2025. Among them, patients who received ward nighttime noise management based on the ERAS protocol during the perioperative period from April 2024 to April 2025 were assigned to the observation group; patients who received conventional postoperative nursing management from April 2023 to March 2024 were assigned to the control group. The two groups were matched at a 1:1 ratio based on the following criteria: gender, age, and ASA classification.

### Ethical consideration:

All procedures performed in this study complied with the ethical standards of the institutional and/or national research committees and the Declaration of Helsinki (revised in 2013). The study was approved by the Medical Ethics Committee of our Hospital (No.: KY20205-149-01; Date: August 8, 2025). Due to the retrospective nature of the study, informed consent was waived by the Medical Ethics Committee of our Hospital. All data were securely stored and remained confidential throughout the study.

### Inclusion Criteria:

- Patients aged 18 years or older.
- Patients with lower extremity fractures who underwent surgical treatment.
- Patients admitted to general wards after surgery.
- Patients with stable vital signs during the perioperative period.
- Complete clinical data.

### Exclusion Criteria:

- Patients complicated with other severe physical diseases, such as cardiopulmonary insufficiency, hepatorenal dysfunction, etc.
- Patients with history of mental illness.
- Patients with history of sleep disorders.
- Patients with hearing impairment.
- Patients with severe postoperative complications.

### Nursing Management Protocols:

### Control Group:

Conventional postoperative nursing management was implemented for the control group:

- Nurses closely monitored patients’ vital signs, wound conditions, as well as blood circulation and swelling of the affected limb. Wound dressing changes, intravenous infusion, and other therapeutic procedures were performed promptly in accordance with medical orders.
- Dietary guidance was provided to patients. Individualized dietary plans were formulated based on patients’ conditions and physical status, encouraging the intake of foods rich in protein, vitamins, and calcium to promote fracture healing.
- Nurses assisted patients with limb function exercises. According to patients’ recovery progress, they guided patients to perform gradual flexion, extension, rotation, and other activities of the lower extremities to prevent muscle atrophy and joint stiffness.
- Psychological support was offered. Nurses proactively engaged with patients to understand their psychological state, promptly addressed their questions and concerns, and encouraged patients to face the disease positively to enhance their confidence in rehabilitation.

### Observation Group:

On the basis of conventional postoperative nursing management, the observation group received nighttime noise management under the ERAS protocol:

- Strengthen ward noise monitoring. Noise monitors were installed in wards. When the noise level exceeded the preset threshold, such as 35 dB, the source was promptly identified, and corresponding control measures were implemented by the night-shift nurse on duty.
- Strengthen medical staff training. Noise management training was delivered yearly by nursing supervisor to medical staff using training brochures, thereby enhancing their awareness of the importance of noise control. The medical staff are assessed annually. Nighttime work norms were formulated, emphasizing the “Four Lights”: speaking softly, walking quietly, operating gently, and closing doors quietly. Unnecessary equipment noise (from multi-parameter ECG monitors, ventilators, electrocardiographs, and bedside call bells) was eliminated, and other noise-generating activities were prohibited. For nursing procedures that had to be performed at night, patients were informed in advance to minimize noise during operation.
- Enhancement of noise management education. Noise management education was delivered by means of lectures for patients and their family members together. Patients were asked to reduce nighttime noise to avoid disturbing other patients in the same ward. Quiet rest areas were provided for accompanying family members to prevent them from wandering and talking freely in the ward.
- Personalized audio environments. Patients were offered personalized audio devices such as headphones or earplugs, allowing them to choose their preferred calming music or natural sounds to reduce external noise interference. The use of headphones or earplugs, soothing music or natural sounds followed standardized criteria.
- Optimization of the ward environment. Sound insulation renovations were carried out on ward doors and windows using soundproof materials to reduce external noise. The ward layout was optimized to separate noise sources (such as, medical equipment storage areas) from patient rest areas, minimizing noise disturbance to patients.

### Primary outcome:

Improvement in sleep quality. The Pittsburgh Sleep Quality Index (PSQI) was used to assess patients’ sleep quality before surgery and seven days after surgery. This scale consists of seven components and 18 items, with each component scored on a range from 0–3 scale. The total PSQI score is the sum of the scores from all components, ranging from 0 to 21, with higher scores indicating poorer sleep quality.

### Secondary outcome:

Psychological state, quality of life, and nursing satisfaction.

*The psychological states include depression and anxiety*. The Self Rating Depression Scale (SDS) was used to assess the depression status of patients. The SDS scale consists of 20 questions, each scored on a scale of 1-4 points. The scores of these 20 items were summed to obtain a raw score; this raw score is then multiplied by 1.25, and the integer part was taken to obtain the standard score. According to the results of the Chinese norm, a standard score below 53 is within the normal range; 53-62 points indicate mild depression; 63-72 points reach moderate depression; Exceeding 73 points is considered severe depression. The Self Rating Anxiety Scale (SAS) was used to assess the level of anxiety in patients. SAS consists of 20 questions, each with a 1-4 level rating. The scores of these 20 projects were summed up to yield a raw score, which is then multiplied by 1.25 to obtain the standard score by taking the integer part. According to the results of the Chinese norm, the cut-off value for SAS standard score is 50 points, with 50-59 points indicating mild anxiety, 60-69 points indicating moderate anxiety, and 70 points or above indicating severe anxiety.^12^

The quality of life is evaluated using the Generic Quality-of-Life Inventory 74 (GQOLI-74), which covers dimensions of material life, psychological function, social function, and physical function. The score for each dimension is one hundred points, and the higher the score, the better the corresponding quality of life.

Nursing satisfaction was evaluated using the Newcastle Nursing Satisfaction Scale, which includes 19 items including nurses’ professional abilities, communication attitudes, psychological counseling, nursing support, and safety management; Each project is scored on a scale of 1-5 points, with a total score of 19-95 points; Among them, ≥ 77 represents “very satisfied”, 58-76 represents “satisfied”, 39-57 represents “moderately satisfied”, and ≤ 38 represents “dissatisfied”; Satisfaction rate=(very satisfied + satisfied + moderately satisfied) / total number of patients. Use SL-4001 noise meter (Shenzhen Shengli High Electronic Technology Co., Ltd.) to measure the noise level. Record nighttime noise levels: daily decibel values from 20:00-20:10 and 22:00-22:10. In this study, the average noise level from April 2023 to March 2024 represents the noise level of the control group, while the average noise level from April 2024 to April 2025 represents the noise level of the observation group.

### Statistical analysis:

The data collected were analyzed using SPSS 26.0 statistical software (SPSS Inc., Chicago, IL, USA), and the figures were plotted using GraphPad Prism-8 (Version 8.3; La Jolla, CA, USA). Firstly, Shapiro-Wilk test was used to test the normal distribution of continuous variables. For normally distributed continuous data (such as noise level), an independent samples t-test was used to compare differences between the two groups, and a paired t-test was applied for intragroup comparisons before and after intervention. Non-normally distributed data were expressed as median and interquartile range (IQR) (such as age, BMI, operation time). The Mann-Whitney U test was used for intergroup comparisons, and the Wilcoxon signed-rank test was employed for intragroup comparisons before and after intervention. The categorical data was presented as frequency (%) (such as gender distribution, education level, ASA classification, and surgical type) and compared using Chi-square test. All statistical tests were two-tailed, with a statistical significance level of 5%.

## RESULTS

**Fig.1:**
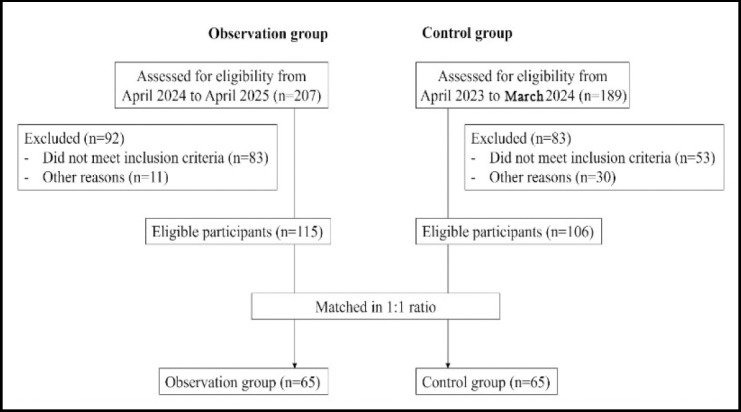
Patient screening flow chart.

From April 2024 to April 2025, the Department of Orthopedics at Gutian Campus of our Hospital implemented ward nighttime noise management based on the ERAS protocol in general wards. During this period, 207 patients were initially selected; 92 patients who did not meet the study criteria were excluded, and 115 patients were enrolled in the observation group. Meanwhile, 189 patients who received conventional nursing management from April 2023 to March 2024 were selected as the control group. After excluding 83 patients who failed to meet the inclusion criteria, 106 patients were enrolled in the control group. The two groups were matched at a 1:1 ratio, resulting in a final sample size of 65 patients per group (Fig.1). As shown in Table-I, there were no statistically significant differences in the baseline characteristics between the two groups (*P* > 0.05); the noise level in the observation group was significantly lower than that in the control group (*P* < 0.05).

**Table-I:** Comparison of baseline characteristics between the two groups.

Characteristics	Observation group (n=65)	Control group (n=65)	Z/t/χ^2^	P
Age (years), median (IQR)	55 (46, 58)	53 (47, 57)	-0.562	0.574
Gender, n(%)			0.493	0.482
Male	32 (49.2)	36 (55.4)		
Female	33 (50.8)	29 (44.6)		
BMI (kg/m^2^), median (IQR)	24.45 (22.03, 27.22)	22.86 (21.25, 25.25)	-1.940	0.052
Educational level, n(%)			0.279	0.597
Junior high school or below	34 (52.3)	37 (56.9)		
Senior high school or above	31 (47.7)	28 (43.1)		
ASA classification, n(%)			0.075	0.784
Grade II	8 (12.3)	7 (10.8)		
Grade III	57 (87.7)	58 (89.2)		
Operation time (minute), median (IQR)	100 (80, 130)	95 (70, 115)	-1.414	0.157
Fracture type, n(%)			6.602	0.086
Femoral fracture	33 (50.8)	33 (50.8)		
Tibial fracture	30 (46.2)	22 (33.8)		
Ankle fracture	1 (1.5)	4 (6.2)		
Calcaneal fracture	1 (1.5)	6 (9.2)		
Noise level (dB), mean±SD	38.9±7.0	48.6±7.6	-7.569	<0.001

***Note:*** BMI, Body mass index; ASA, American Society of Anesthesiologists; SD, Standard deviation; IQR, Interquartile range.

**Fig.2:**
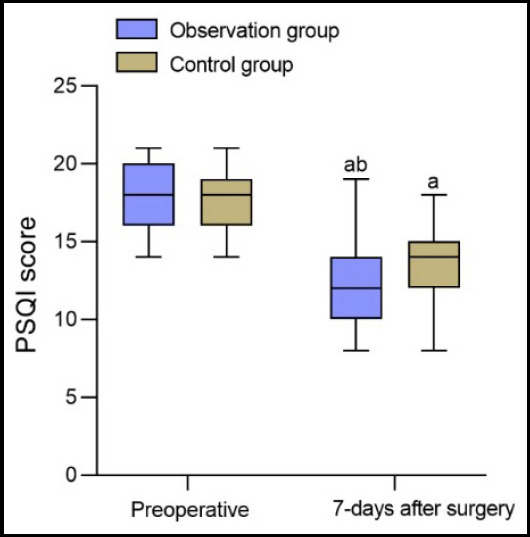
Comparison of PSQI scores between the two groups. Compared with the preoperative period in the same group, ^a^P < 0.05; compared with the control group, ^b^P < 0.05.PSQI, Pittsburgh Sleep Quality Index.

There were no statistically significant differences in the preoperative PSQI scores between the two groups (*P* > 0.05). Seven days after surgery, the PSQI scores of both groups were significantly lower than those before surgery, and the score in the observation group was significantly lower than that in the control group (*P* < 0.05) (Fig.2).

**Fig.3:**
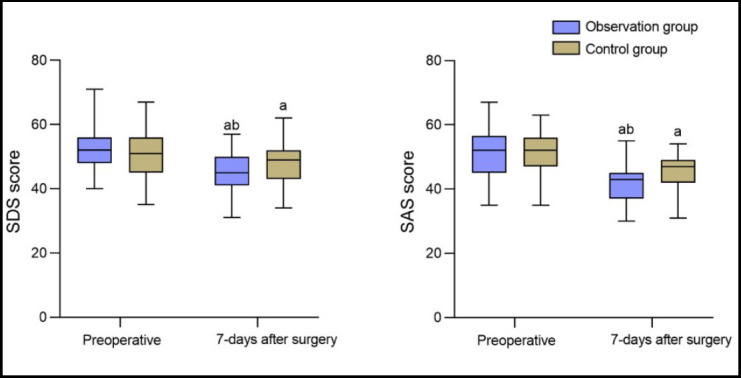
Comparison of SDS and SAS scores between the two groups. Compared with the preoperative period in the same group, ^a^P < 0.05; compared with the control group, ^b^P < 0.05.SDS, Self-Rating Depression Scale; SAS, Self-Rating Anxiety Scale.

Before surgery, there were no statistically significant differences in the SDS and SAS scores between the two groups (*P* > 0.05). Seven days after surgery, the SDS and SAS scores of both groups were lower than those before surgery, and the scores in the observation group were significantly lower than those in the control group (*P* < 0.05) (Fig.3).

**Fig.4:**
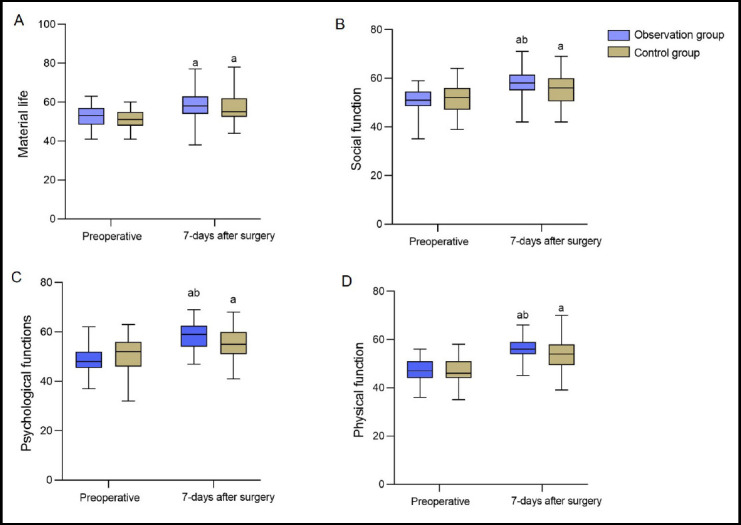
Comparison of GQOLI-74 scores between two groups of patients. A. Comparison of material life scores; B. Comparison of social function scores; C. Comparison of psychological function scores; D. Comparison of physical function scores.. Compared with the preoperative period in the same group, ^a^P < 0.05; compared with the control group, ^b^P < 0.05.

Similarly no statistically significant differences were observed in the scores of all dimensions of the GQOLI-74 between the two groups (*P* > 0.05). Seven days after surgery, the scores of all domains in both groups were higher than those before surgery, the scores of social function, psychological function, and physical function in the observation group were significantly higher than those in the control group (*P* < 0.05). But there was no statistically significant difference in the material life score between the two groups (*P* > 0.05) (Fig.4).

The comparison of nursing satisfaction between the two groups showed that the nursing satisfaction of the observation group was 93.8%, which was significantly higher than that of the control group (81.5%) (*P* < 0.05) (Table-II).

**Table-II:** Comparison of nursing satisfaction between the two groups.

Group	Very satisfied	Satisfied	Moderately satisfied	Dissatisfied	Satisfaction rate
Observation group (n=65)	38 (58.5)	14 (21.5)	9 (13.8)	4 (6.2)	61 (93.8)
Control group (n=65)	24 (36.9)	18 (27.7)	11 (16.9)	12 (18.5)	53 (81.5)
*χ^2^*					4.561
*P*					0.033

## DISCUSSION

This was a retrospective, non-randomized, historically controlled study conducted in the Department of Orthopedics, Gutian Campus of our Hospital. Since April 2024, our department has implemented nighttime noise management based on the ERAS protocol in general wards, formulating relevant standardized regulations for medical staff, patients, and their families. Noise reduction optimization was also carried out for the ward environment. The results showed that the ward noise level was significantly lower than that of the previous year (38.9±7.0 vs. 48.6±7.6 dB). We selected two cohorts of lower limb fracture patients who were in different noise environments and received surgical treatment. The results of this study suggest that nighttime noise management under the ERAS protocol was associated with better postoperative sleep quality, mental health, and quality of life, which is consistent with previous relevant studies. Wang et al.^13^ compared two groups of lung cancer patients with significantly different noise environments, demonstrating that nighttime noise management under the ERAS background can improve patients’ sleep quality and mental health. Chen et al.^11^ also confirmed that ward noise management helps alleviate anxiety in elderly patients after total hip arthroplasty, improves their quality of life and social function, and achieves higher satisfaction. It can be seen that nighttime noise management based on the ERAS protocol provides a good ward environment for patients and plays a key role in improving their sleep and mental state.

The study also showed that the sleep quality of the observation group was better than that of the control group after surgery, which indicates that reducing the nighttime noise level in the ward through noise management measures enabled patients to fall asleep faster, increased sleep depth, and improved sleep efficiency, thereby enhancing their sleep quality. This is consistent with the research results of Zhang et al.^14^ A favorable sleep environment can reduce the frequency of nocturnal awakenings, prolong the duration of deep sleep, and improve sleep quality.^14^,^15^ Meanwhile, good sleep is conducive to wound healing and the repair of fractured sites. However, we also need to acknowledge that poor preoperative sleep quality may be associated with anxiety while awaiting surgery and fracture instability, while improved postoperative sleep quality may be associated with the physiological relief provided by fracture fixation and analgesia after surgery.

Patients with lower limb fractures are prone to adverse emotions such as depression and anxiety.^10^,^11^,^16^ Studies have shown that patients’ emotional states and anxiety levels can affect their sleep quality and exert adverse effects on physical rehabilitation.^16^,^17^ In this study, the SDS and SAS scores of the observation group were lower than those of the control group after the implementation of ward nighttime noise management. This is because a quiet and comfortable ward environment makes patients feel more relaxed and reduces external stimuli that trigger adverse emotions, which may contribute to bolstering their confidence in recovery or improving treatment adherence. Palagini et al.^18^ also confirmed that good sleep and a quiet environment can reduce the level of stress hormones in patients, alleviate anxiety and depression symptoms, and promote mental health. In addition, we provided psychological counseling to patients and their families to enhance their confidence in treatment and psychological coping abilities. The current findings suggest an association between comprehensive nighttime quiet management and better psychological outcomes, but do not establish noise reduction alone as the sole or dominant mechanism. Psychological improvement may have been achieved through a combination of better sleep, lower environmental stress, and enhanced care experience.

In addition, this study found that in terms of material life, the postoperative scores of both groups improved, but there was no significant difference between the two groups. We attribute primarily to the standardized material support and rehabilitation exercise guidance provided by the hospital to postoperative patients. In terms of social function, psychological function and physical function, the scores in the observation group were all higher than those in the control group. This was closely related to the improvement in patients’ sleep quality and mental health resulting from noise management. Good sleep and a positive psychological state accelerated physical recovery, alleviated pain perception, and enabled patients to participate in social activities more actively. With sufficient sleep and a pleasant mood, patients were more willing to communicate and interact with others, and their social ability as well as physical and mental health were restored.^19^ These findings are consistent with those reported by Huang et al.^19^ The improvement in social function, psychological function and physical function is of great significance for the comprehensive rehabilitation of patients. It not only facilitates psychological recovery but also improves patients’ quality of life and sense of self-identity.^19^–^21^ In addition, the satisfaction rate of patients in the observation group (93.8%) was significantly higher than that in the control group (81.5%). This indicates that nighttime noise management allowed patients to obtain adequate sleep, recover physical strength better, and alleviate physical fatigue; consequently, they provided positive evaluations of their hospitalization experience, thereby improving their overall satisfaction.^22^,^23^

### Strengths:

This study provides clinical evidence that nighttime noise management based on the ERAS protocol may be associated with improved sleep quality, mental health, and quality of life in patients with lower extremity fractures. Furthermore, it also verifies that noise management constitutes a crucial component in enhancing the quality of hospital services and management. Besides sound control, behavioral regulation, adjustment of nursing rhythm, caregiver management, patient comfort support, and ward order maintenance were also implemented. Nighttime ward environment management may serve as part of an integrated recovery-promoting care pathway under ERAS. However, in this study, the noise level of the observation group (38.9±7.0 dB) was still higher than the 30 dB nighttime noise level recommended by the WHO. It can be seen that there are still certain challenges in noise management.

### Limitations:

Firstly, it is a single-center retrospective analysis with a small sample size, and the samples were selected within a specific time period. Therefore, the generalizability of the research results may be limited. Future prospective, multi-center, large-sample studies are needed to confirm whether the conclusions of this study are applicable to other orthopedic populations or non-orthopedic populations with different pain profiles and nighttime care needs. Secondly, regarding noise management measures, some measures may not be fully implemented. For example, there is a lack of quantitative evaluation indicators for supervising the implementation of the “Four Lights” principle among medical staff, making it difficult to accurately judge the degree and effect of implementation. Other process indicators such as training coverage, earplug usage rate, or adherence to nighttime noise management standards were not analyzed. Thirdly, factors such as patients’ cultural backgrounds and family conditions may have certain interference with the research results. Finally, it is necessary to optimize noise management measures to further reduce the nighttime noise level in the ward. At the same time, multi-center, large-sample randomized controlled trials are required to enhance the representativeness and reliability of the results of this study.

## CONCLUSION

The results of this study suggest that ward nighttime noise management under the ERAS protocol was associated with better postoperative recovery-related outcomes in patients with lower extremity fractures. Nighttime noise management is associated with improved patients’ sleep quality and mental health, enhancing their quality of life, and achieve high nursing satisfaction. Nighttime ward environment management may represent a relatively low-cost, integrable nursing optimization strategy within ERAS pathways and may be worth further exploration in orthopedic general wards, which provides important practical guidance for clinical nursing work. However, high-quality randomized controlled studies are still needed to verify the reliability and universality of the conclusions.

### Author’s contributions:

**RZ:** Literature search, study design and manuscript writing.

**RZ and CH:** Data collection, data analysis and interpretation. Critical Review.

**RZ:** Manuscript revision and validation and is responsible for the integrity of the study.

All authors have read and approved the final version of the manuscript.
